# A nationwide mobile phone survey for tobacco use in Tanzania: Sample quality and representativeness compared to a household survey

**DOI:** 10.1016/j.pmedr.2024.102609

**Published:** 2024-01-12

**Authors:** Gulam Muhammed Al Kibria, Frank Kagoro, George Pariyo, Joseph Ali, Farida Hassan, John W. Kilambo, Irene Petro, Vidhi Maniar, Michelle R. Kaufman, Andres Vecino-Ortiz, Saifuddin Ahmed, Honorati Masanja, Dustin G. Gibson

**Affiliations:** a Department of International Health, Johns Hopkins Bloomberg School of Public Health, Baltimore, MD 21205, United States; b Ifakara Health Institute, Dar es Salaam, Tanzania; c Center for Optimising Antimalarial Therapy, University of Cape Town, Cape Town, South Africa; d The Global Health Network, Center for Tropical Medicine and Global Health, University of Oxford, Oxford, United Kingdom; e Serum Africa Medical Research Institute, Kampala, Uganda; f Berman Institute of Bioethics, Johns Hopkins University, Baltimore, MD 21205, United States; g Department of Health, Behavior and Society, Johns Hopkins Bloomberg School of Public Health, Baltimore, MD 21205, United States; h Department of Population, Family & Reproductive Health, Johns Hopkins Bloomberg School of Public Health, Baltimore, MD 21205, United States

**Keywords:** Mobile phone survey, Survey findings, Interactive voice response, Tobacco use, Data collection

## Abstract

We investigated the feasibility of an interactive voice response (IVR) survey in Tanzania and compared its prevalence estimates for tobacco use to the estimates of the 'Global Adult Tobacco Survey (GATS) 2018′. IVR participants were enrolled by random digit dialing. Quota sampling was employed to achieve the required sample sizes of age-sex strata: sex (male/female) and age (18–29-, 30–44-, 45–59-, and ≥60-year-olds). GATS was a nationally representative survey and used a multistage stratified cluster sampling design. The IVR sample’s weights were generated using the inverse proportional weighting (IPW) method with a logit model and the standard age-sex distribution of Tanzania. The IVR and GATS had 2362 and 4555 participants, respectively. Compared to GATS, the unweighted IVR sample had a higher proportion of males (58.7 % vs. 43.2 %), educated people (secondary/above education: 43.3 % vs. 21.1 %), and urban residents (56.5 % vs. 40 %). The weighted prevalence (95 % confidence interval (CI)) of current smoking was 4.99 % (4.11–6.04), 5.22 % (4.36–6.24), and 7.36 % (6.51–8.31) among IVR (IPW), IVR (age-sex standard), and GATS samples, respectively; the weighted prevalence (95 % CI) of smokeless tobacco use was similar: 3.54 % (2.73–4.57), 3.58 % (2.80–4.56), and 2.43 % (1.98–2.98), respectively. Most differences in point estimates for tobacco indicators were small (<2%). Overall, the odds of tobacco smoking indicators were lower in IVR than in GATS; however, the odds of smokeless tobacco use were reversed. Although we found under-/over-estimation of the prevalence of tobacco use in IVR than GATS, the estimates were close. Further research is required to increase the representativeness of IVR.

## 1 Introduction

Globally, noncommunicable diseases (NCDs), such as cancer, heart disease, and diabetes, are the leading causes of death (Dicker et al., 2018). Over the past three decades, the number of deaths, years of life lost, years lived with disability, and disability-adjusted life years attributable to these conditions have increased in low- and middle-income countries (LMICs) (Dicker et al., 2018, Forouzanfar et al., 2016, Wang et al., 2016). Many LMICs are now dealing with a double disease burden – a simultaneous high burden of communicable and noncommunicable diseases (Dicker et al., 2018). Tobacco consumption is a major modifiable behavioral risk factor for these diseases; its use increases the risks for cancer, cardiovascular, and respiratory disease (U.S. Department of Health and Human Services, 2014). More than 8 million deaths are caused by tobacco use globally each year (Reitsma et al., 2017).

Continuously monitoring the burden of diseases and risk factors of public health importance, such as tobacco use, helps develop effective programs and policies to reduce their future burden (World Health Organization, 2021). Currently, the World Health Organization’s STEPwise approach to NCD Risk Factor Surveillance (WHO STEPS), Multiple Indicator Cluster Survey (MICS), Demographic and Health Surveys (DHS), and Global Adult Tobacco Surveys (GATS) are used to obtain nationally representative health data from several countries (United States Agency for International Development, 2022, World Health Organization, 2022). However, conducting face-to-face interviews to collect such data is expensive, time-consuming, and labor-intensive. The challenge of cost and effort may affect the ability to collect survey data and, therefore, impact efforts to reduce the burden of NCD (Blinson et al., 1996, DeFranzo, 2021).

In high-income countries (HICs), telephone interviews are often conducted to collect data on behavioral risk factors. For example, the Behavioral Risk Factor Surveillance System (BRFSS) collects U.S. residents’ data on health risk behaviors, chronic conditions, and preventive service use (Centers for Disease Control and Prevention, 2017). Lack of telephone access was previously an obstacle to implementing such surveillance in LMICs; however, the increasing use of mobile phones globally may allow for implementing mobile phone surveys (MPS) in LMICs. More than 95 % of people globally use mobile phones (International Telecommunication Union, 2018). In most LMICs, a majority of people live in rural regions, and the distance from one geographic location to another is large; therefore, MPS could be more useful for collecting data from these hard-to-reach population groups (L’Engle et al., 2018, Leo et al., 2012).

Multiple MPS data collection methods have been developed (Ballivian et al., 2015, Gibson et al., 2017). Interactive voice response (IVR) is an MPS method where eligible participants use their mobile phone keypad to answer a prerecorded questionnaire through an automated system (e.g., “If you are a male, press 1. If you are a female, press 2”). IVR has been used to collect nationally representative estimates of demographic and health indicators (Song et al., 2020). However, the representativeness, reliability, or how the prevalence estimates reported by IVR differ from the nationally representative data collected by household face-to-face surveys, the gold standard, have not been well studied in many countries. The validity of indicators reported by MPS or how they are similar to those reported by the household face-to-face surveys are unknown. The United Republic of Tanzania is an example of an LMIC that is currently dealing with the double disease burden. This is a sub-Saharan African country with a population of about 60 million. In 2020, the mobile phone subscription rate was 86 per 100 people (The World Bank, 2023). An IVR was conducted in this country to understand the feasibility and cost of conducting a nationally representative survey. In this study, we attempt to compare the representativeness and validity of IVR data by comparing its tobacco use estimates with GATS Tanzania 2018 data.

## 2 Methods

### 2.1 Study design and participants

We conducted a cross-sectional study here. The IVR participants were recruited by random digit dialing (RDD*)*. RDD sampling is a probability sampling technique where a software system generates a list of phone numbers at random to be used as the sampling frame. The sample was drawn from the RDD sampling frame by calling the participants (Research, 2023, Waksberg, 1978). Quota sampling was used to recruit participants of the following age-sex strata: 18–29-, 30–44-, 45–59-, and ≥60-year-old males and females. Due to the RDD, we did not have a specific sampling frame in the IVR. The method of obtaining the sample was described in the procedure section.

The GATS Tanzania 2018 was a part of the Global Tobacco Surveillance System. This nationally representative household survey aimed to estimate tobacco use indicators among ≥15-year-old non-institutional people. A standard survey protocol with standardized questionnaires, sample design, data management, and analysis procedures were followed. Data were collected using a multistage (i.e., three-stage) cluster sampling design to report estimates for the country as a whole and males and females of rural and urban regions. To make the survey sample nationally representative and to provide reliable national estimates for tobacco use indicators, GATS 2018 covered all regions (i.e., 31 areas −26 from Mainland and 5 from Zanzibar) of Tanzania. The Population and Housing Census of 2012 was used as the sampling frame. The sampling frame had the lists of regions, districts, wards, and enumeration areas. In the first stage, 84 urban and 120 rural clusters were selected. In the second stage, 26 households were randomly chosen from the household list in each cluster. At last, one person with at least 15 years of age from each household was randomly interviewed (Ministry of Health, Community Development, Gender, Elderly and Children Dodoma et al., 2020).

The questionnaire primarily asks about sociodemographic characteristics, tobacco smoking, and smokeless tobacco use, among others (Ministry of Health, Community Development, Gender, Elderly and Children Dodoma et al., 2020). The GATS sampling frame included all households in a cluster. From each sampled household (5297), one participant with at least 15 years of age was selected. This resulted in a total of 4797 respondents, with a 96.4 % individual response rate. Data were collected from February to April 2018 (Ministry of Health, Community Development, Gender, Elderly and Children Dodoma et al., 2020).

### 2.2 Procedures

Data collection for IVR took place from October 2020 to March 2021. The phone calls were administered between 8:00 AM and 8:00 PM local time and sent in Kiswahili. Only randomly generated numbers were called. For RDD, the first three digits of the phone numbers were the country code, the next three digits were the mobile network operator’s base digits, and the remaining seven digits were randomly generated to create a mobile phone number. These included all the existing Tanzanian mobile operators (i.e., 8). Upon answering the phone, participants were told about the purpose of the study, its expected duration and sponsoring agency, and the requirements for receiving an airtime survey. Participants were eligible if they were at least 18 years old and the sample size for the age-sex strata to which they belonged had not been met. Eligible participants were read a brief consent statement and asked to press ‘1′ if they consented to participate.

The IVR survey had five major components: 1) survey introduction, 2) age-sex screening questions, 3) consent, 4) demographic questions, and 5) five NCD modules. The NCD modules included questions related to 1) tobacco use, 2) alcohol consumption, 3) dietary habits, 4) physical activity, and 5) blood pressure and diabetes. The order of NCD modules was also randomly assigned for each participant to reduce attrition bias. However, the questions were not randomized within each module to preserve skip patterns. Participants who completed the survey were entered into a lottery where 1 in 20 complete surveys would receive 50,000 Tanzanian Schillings worth of mobile phone airtime (USD 21.68 as of October 20, 2020). Participants were also informed that they would not need to pay any money for the survey.

### 2.3 Outcomes

We limited our analysis to participants with completed IVR interviews. An interview was considered complete (I) when the participants answered at least four of the five NCD modules. Interviews with one to three modules completed were considered partial interviews (P). Refusals (R) were considered when age-eligible participants did not indicate consent or terminate the survey before consenting. Age-eligible participants who consented but did not complete any NCD module were considered break-offs (R). Those who did not answer the age question after initiating the survey were unknown (U). The estimated proportions of age-eligible respondents (e) were selected from people screened for age-eligibility but remained of unknown status. Individuals who indicated they were not at least 18 years old were considered age-ineligible. The response and cooperation rates were calculated using the American Association for Public Opinion Research equations (The American Association for Public Opinion Research., 2016) (S1 Table).

We compared indicators that were available in both surveys. The primary outcomes were responses related to tobacco use: current smoking, current daily smoking, past smoking, past daily smoking, current smokeless tobacco use, current daily smokeless tobacco use, former smokeless tobacco use, and former daily smokeless tobacco use. The proportion of current smokers or smokeless tobacco users was obtained by dividing the number of participants who responded ‘yes’ to that question by the number of participants who responded to that question. Skip patterns of questionnaires were considered to calculate this. At the end of the survey, participants were asked how satisfied they were with the survey.

The questions are presented in the S2 Table. In GATS, the current smokers were those who smoked tobacco currently (i.e., daily or less than daily); the former and former daily smokers were those who smoked tobacco in the past. Then, current and past smokeless tobacco users were those who used smokeless tobacco currently and in the past (i.e., daily or less than daily), respectively. IVR asked the questions directly (S2 Table).

### 2.4 Sample size calculation

The IVR’s sample size was calculated for eight age-sex strata (i.e., 18–29-, 30–44-, 45–59-, and ≥60-year-old male and female respondents) and was obtained based on the assumptions of 50 % prevalence rate (p = 0.5), 5 % type 1 error (α = 0.05) and margin-of-error (*δ* = 0. 05). For each stratum, 385 complete surveys were required, with a total of 3080 for all eight strata.

### 2.5 Ethics approval

The study received ethical approval from the Institutional Review Boards of Johns Hopkins Bloomberg School of Public Health, Baltimore, MD, USA, and Ifakara Health Institute, Dar es Salaam, Tanzania.

### 2.6 Statistical analysis

There were methodological differences between IVR and GATS, including the age requirement and quota sampling. We limited our analyses to people at least 18 years old and applied ‘weighting’ to the IVR sample to minimize these.

Weighting was also required to reduce the proportion of sociodemographic differences between GATS and IVR participants. We used two weighting methods to generate the IVR sample’s weight. First, we employed a logistic regression model to generate inverse proportional weights (IPW), considering the GATS as the reference. This creates a sample weight for each of the participants of GATS. We adjusted for age, sex, education, and location of residence to generate these weights. Then, we used the United Nations Department of Population’s standard age-sex distribution for Tanzania to get the sample weight for age-sex strata (United Nations, 2019).

To understand how these samples differed, first, we described the unweighted and weighted sociodemographic characteristics of GATS and IVR participants. Then, we reported unweighted and weighted prevalence (with 95 % confidence intervals [CI]). Lastly, using the weighted GATS sample as the reference, we conducted unadjusted and adjusted logistic regression analyses to test the association of survey mode with tobacco use indicators. We adjusted indicators for age, sex, education, and location, and reported crude and adjusted odds ratio (OR) with 95 % CI. We calculated the direct delivery cost per complete survey for each age-sex strata, which included the cost of airtime used to complete the survey and the incentive. The time spent answering the survey was multiplied by the per-minute airtime cost. Data analyses were conducted using Stata 14.1 (Stata Corporation, College Station, Texas USA, 2017).

## 3 Results

**Fig. 1:**
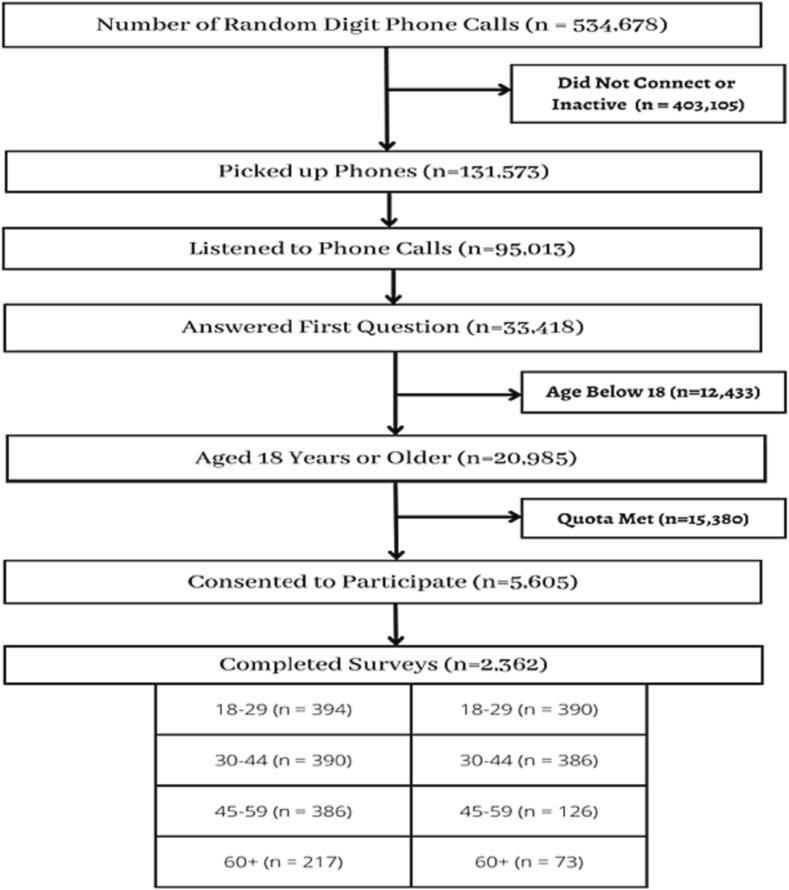
Flow Diagram of the Retention of the Interactive Voice Response Survey’s Study Sample, Tanzania.

A total of 534,678 IVR calls were made; 20,985 respondents indicated they were at least 18 years old, and 5605 consented to participate. The number of completed interviews was 2362 (Fig. 1). Sample sizes for five of the eight age-sex strata were reached: 18–29- and 30–44-year-old males and females, and 45–59-year-old males. The sample sizes for unfilled age-sex strata for females ages 45–59, males ages 60+, and females ages 60+ were 126, 217, and 73, respectively. The cost per complete survey increased with age (S3 Table).

The disposition codes and participation rates are reported in Table 1. The contact, response, cooperation, and refusal rates were 1.3 %, 0.8 %, 46.4 %, and 0.5 %, respectively. Most respondents were satisfied with the survey (98.4 %).

**Table 1:** Description of the Participants Disposition Codes and Participation Rates of the Interactive Voice Response Survey (N = 534,768), Tanzania 2020–21.

Variable	% (N)
**Interview Type**	
Complete interview (I)	0.4 (2362)
Partial Interview (P)	0.3 (1717)
Breaks-off (R)	0.3 (1526)
Refusal (R)	0.2 (1208)
Ineligible: Age < 18	1.1 (6074)
Unknown age and/or sex not provided (U)	19.7 (105,473)
Did not pick up or line not connected (U)	75.4 (403,186)
Ineligible: quota met	2.5 (13,222)

**Survey rates**	
Contact rate#1	1.3 (6813/515,472)
Response Rate #2	0.8 (4079/515,472)
Cooperation rate #1	46.4 (2362/5096)
Refusal rate #1	0.5 (2734/515,472)
Satisfaction rate	98.4 (2235/2271))

Table 2 shows the sociodemographic characteristics by survey mode. The proportions of 18–29-, 30–44-, 45–59-, and ≥60-year-olds were 33.2 %, 32.9 %, 22.0 %, and 12.0 %, respectively, in IVR; these proportions were 33.0 %, 35.5 %, 16.7 %, and 14.8 %, respectively, in GATS. The proportions of males in IVR and GATS were 41.3 % and 56.5 %, respectively. Overall, the proportion of people with secondary or higher education was higher in IVR than in GATS, at 43.3 % and 21.1 %, respectively. The proportion of urban residents was 56.5 % in IVR, while this was 39.9 % in GATS. The age-sex distribution of IVR and GATS samples became similar after weighting. The age-sex weighted IVR sample (58.7 %) had a higher proportion of urban residents than the IPW IVR (38.9 %) and GATS (33.2 %) samples. The proportion of people with any formal education was also higher among the age-sex weighted IVR sample (93.6 %) compared to the IPW IVR (84.6 %) and GATS (83.9 %) weighted samples.

**Table 2:** Comparison of IVR & GATS Sample to Study Participants’ Sociodemographic Characteristics, Tanzania.

Variable		Unweighted, % (N)		Weighted, %		
		IVR	GATS	IVR (IPW Method)	IVR (Age-Sex Standard Pop)	GATS
Age (in year)	18–29	33.2 (784)	33.0 (1503)	43.8	41.3	41.6
	30–44	32.9 (776)	35.5 (1617)	34.1	33.1	32.3
	45–59	22.0 (519)	16.7 (762)	14.6	17	16.4
	60+	12.0 (283)	14.8 (673)	7.5	8.5	9.7
Sex	Male	41.3 (975)	56.5 (2708)	49.6	50.6	52.7
	Female	58.7 (1387)	43.5 (2089)	50.4	49.4	47.3
Education Level	No School	7 (165)	17.7 (847)	15.4	6.4	16.1
	Primary	49.7 (1174)	61.3 (2935)	61.6	49.2	61.5
	Secondary	29.7 (700)	17.5 (837)	18.9	30.8	19.1
	Tertiary	13.6 (321)	3.6 (171)	4.2	13.6	3.3
Location	Urban	56.5 (1330)	39.9 (1914)	38.9	58.7	33.6
	Rural	43.5 (1024)	60.1 (2883)	61.1	41.3	66.4

Abbreviations: GATS: Global Adult Tobacco Survey, IPW: Inverse Proportion Weight, IVR: Interactive Voice Response.

Table 3 shows the unweighted and weighted prevalence of tobacco indicators. The weighted prevalence (95 % CI) of current smoking was 4.99 % (4.11–6.04), 5.22 % (4.36–6.24), and 7.36 % (6.51–8.31) among IPW IVR, age-sex weighted IVR, and GATS respondents, respectively; the prevalence (95 % CI) of current daily smoking was 3.12 % (2.47–3.93), 3.10 % (2.50–3.84), and 5.67 % (4.93–6.52), respectively. Most other IVR prevalence estimates were close to GATS estimates. After stratifying the unweighted sample by age and sex, the overall proportion of current smokers or current daily smokers was lower among IVR respondents than their GATS counterparts, and most differences were insignificant (S4 and S5 Tables).

**Table 3:** Comparison of Unweighted and Weighted Prevalence (95 % CI) of Tobacco Use Indicators among GATS and IVR Participants, Tanzania.

Indicators	Unweighted		Weighted		
	IVR	GATS	IVR (IPW Method)	IVR (Age-Sex Standard Pop)	GATS
Current smoker	6.45(5.53,7.52)	7.88(7.15,8.68)	4.99(4.11,6.04)	5.22(4.36,6.24)	7.36(6.51,8.31)
Current daily smoker	4.38(3.62,5.28)	6.21(5.56,6.93)	3.12(2.47,3.93)	3.10(2.50,3.84)	5.67(4.93,6.52)
Former smoker	6.93(5.97,8.03)	5.36(4.75,6.03)	5.83(4.84,7.01)	5.70(4.81,6.74)	5.12(4.40,5.96)
Former daily smoker	3.23(2.59,4.03)	3.06(2.61,3.59)	2.49(1.89,3.27)	2.31(1.80,2.96)	2.82(2.30,3.45)
Current smokeless tobacco user	3.52(2.85,4.34)	2.92(2.48,3.44)	3.54(2.73,4.57)	3.58(2.80,4.56)	2.43(1.98,2.98)
Current daily smokeless tobacco user	1.74(1.28,2.35)	2.17(1.80,2.63)	1.65(1.15,2.37)	1.69(1.17,2.42)	1.73(1.35,2.21)
Former smokeless tobacco user	3.10(2.47,3.88)	1.92(1.57,2.35)	2.86(2.18,3.76)	2.72(2.11,3.49)	1.62(1.24,2.12)
Former daily smokeless tobacco user	0.93(0.62,1.41)	0.84(0.61,1.14)	0.87(0.55,1.37)	0.87(0.56,1.36)	0.60(0.40,0.88)

Abbreviations: CI: Confidence Interval, IVR: Interactive Voice Response, GATS: Global Adult Tobacco Survey.

Table 4 shows the odds of reporting indicators comparing the unweighted and weighted IVR and GATS estimates. Overall, compared to the GATS sample, the odds were lower for smoking variables but higher for smokeless tobacco variables among IVR samples. For instance, compared to the GATS sample, among unweighted IVR, IPW IVR, and age-sex standard IVR, the AOR (95 % CI) of current smoking was 0.59 (0.48–0.73), 0.58 (0.58,-0.58), and 0.75 (0.62–0.91), respectively; on the other hand, the AOR (95 % CI) for smokeless tobacco users was 1.59 (1.16–2.16), 1.68 (1.68–1.69), and 2.29 (1.83–2.86), respectively.

**Table 4:** Unadjusted and Adjusted Odds Ratios (95 % CI) for the Association Tobacco Use Indicators with Survey Modes, Tanzania.

Indicators	Unweighted		Weighted			
			IVR (IPW Method) vs GATS (Ref)		IVR (Age-Sex Standard Pop) vs GATS (Ref)	
	Unadjusted	Adjusted	Unadjusted	Adjusted	Unadjusted	Adjusted
Current smoker	0.77** (0.63,0.93)	0.59*** (0.48,0.73)	0.66*** (0.66,0.66)	0.58*** (0.58,0.58)	0.69*** (0.58,0.83)	0.75** (0.62,0.91)
Current daily smoker	0.66*** (0.52,0.82)	0.48*** (0.37,0.62)	0.53*** (0.53,0.54)	0.45*** (0.45,0.45)	0.53*** (0.42,0.67)	0.54*** (0.43,0.69)
Former smoker	1.25* (1.02,1.53)	0.97 (0.78,1.21)	1.15*** (1.14,1.15)	1.08*** (1.07,1.08)	1.12 (0.94,1.33)	1.10 (0.92,1.32)
Former daily smoker	1.00 (0.76,1.33)	0.80 (0.59,1.08)	0.88*** (0.88,0.88)	0.80*** (0.80,0.81)	0.81 (0.62,1.07)	0.83 (0.63,1.09)
Current smokeless tobacco user	1.16 (0.88,1.52)	1.59** (1.16,2.16)	1.47*** (1.47,1.47)	1.68*** (1.68,1.69)	1.49*** (1.20,1.85)	2.29*** (1.83,2.86)
Current daily smokeless tobacco user	0.76 (0.53,1.10)	1.20 (0.79,1.82)	0.96*** (0.95,0.96)	1.19*** (1.19,1.20)	0.97 (0.71,1.33)	1.85*** (1.34,2.56)
Former smokeless tobacco user	1.55** (1.13,2.11)	1.54* (1.10,2.16)	1.79*** (1.78,1.79)	1.73*** (1.73,1.74)	1.69*** (1.32,2.17)	1.90*** (1.48,2.45)
Former daily smokeless tobacco user	1.06 (0.63,1.79)	1.45 (0.81,2.58)	1.45*** (1.45,1.46)	1.41*** (1.40,1.42)	1.47 (0.95,2.27)	2.52*** (1.63,3.92)

1. Adjusted for age, sex, education, and location; *: p < 0.5, **: p < 0.01. ***: p < 0.001

Abbreviations: CI: Confidence Interval, IVR: Interactive Voice Response, GATS: Global Adult Tobacco, IPW: Inverse Proportion Weight.

## 4 Discussion

In this study, we employed RDD and quota sampling to determine the feasibility of using an IVR survey to collect nationally representative data in Tanzania and compared the prevalence estimates of tobacco use indicators using two different survey modes (i.e., IVR and face-to-face). We found a significant difference in the likelihood of reporting these indicators; overall, the odds of reporting tobacco smoking indicators were lower, and smokeless tobacco use indicators were higher among IVR respondents compared to those of GATS; however, most of the prevalence estimates were close to one another (i.e., a < 2 % difference). This study adds to a growing body of literature investigating MPS's usefulness and validity in LMICs.

Different evaluation modes can generate different prevalence estimates (Clagett et al., 2013, Worges et al., 2022). In many HICs, multiple surveys are conducted each year to obtain prevalence estimates. Several studies examined the differences in prevalence estimates according to survey mode (Carlson et al., 2009, Hsia et al., 2020, Keadle et al., 2016). For instance, Keadle and colleagues examined the prevalence of physical activity (PA) among older individuals using the National Health and Nutrition Examination Survey (NHANES), National Health Interview Survey (NHIS), and BRFSS data; NHANES and NHIS were in-person surveys while BRFSS was a telephone-based survey [23]; they found the estimates of meeting PA guidelines as 27 %, 36 %, and 44 %, respectively (Keadle et al., 2016). Despite these differences in estimates, all three data collection methods are in use. Additionally, as the IVR and GATS used different sets of questions for the same indicators, differences in the wording of questions can yield different estimates (Carlson et al., 2009, Hsia et al., 2020, Keadle et al., 2016).

Although we found differences in the odds of reporting different tobacco use indicators, the point estimates were close to one another. We observed similar differences after stratifying the unweighted sample by age and sex (S4 and S5 Tables). The small differences in point estimates (<2%) of most tobacco use indicators between these two survey modes indicate that IVR can obtain similar estimates as other surveys. The observed differences can also arise from the differences in the study sample (i.e., age, sex, education, and location) and variability in the timing of survey administration. The IVR sample had a relatively higher proportion of people with higher education and urban residence. These population groups are also more likely to use and own cellular devices in LMICs (Greenleaf et al., 2019, Poirier et al., 2021), and previous studies have shown differences in tobacco use by these characteristics (Reitsma et al., 2017, Sreeramareddy and Acharya, 2021, Tingum et al., 2017). The under- or over-representation of some population groups is common in MPS studies. For instance, a previous study by L’Engle and colleagues compared the findings of an RDD IVR survey with the Ghana DHS. Although the results of that study were promising, and they enrolled a large sample in a short period, they also had an underrepresentation of women and rural and older people (L’Engle et al., 2018). Another study by Greenleaf et al. in Burkina Faso found about two times higher odds of reporting modern contraceptive use among RDD women compared to data from women obtained using face-to-face interviews (Greenleaf et al., 2020).

We could not achieve sufficient sample sizes for 45–59- and 60+ -year-old females (S5 Table). In addition to lower ownership of mobile phones, females in LMICs tend to spend more time doing household chores and caring for children, which may prevent them from answering phone calls (L’Engle et al., 2018). Future MPS should explore ways to reach women and other underrepresented population groups (i.e., elderly, less educated, and rural residents), such as varying the timeframe in which calls are made or having the respondent suggest a better time for outreach. The average cost and number of calls for a survey completed by a woman were also higher than that of men because of our quota sampling approach. The average cost for recruiting younger people was lower than household surveys; however, this increased substantially with increasing age (S3 Table). More research is required to understand the methods to minimize IVR costs. After stratification by age and sex, we could not obtain some estimates due to the smaller sample size of some age-sex groups (e.g., 60+-year-olds, S5 Table). Previous studies have shown that airtime incentives increase participation rates (Gibson et al., 2019). Although we used incentives in this survey, one possible solution is to oversample individuals from underrepresented or hard-to-reach population groups. Other nationally representative surveys (e.g., NHANES) also follow that approach and generate the sample weight after data collection to reflect population-level estimates. Though we used quota sampling to obtain age-sex strata, additional strata like age-sex-education-place of residence could be used. Other strategies could be to use motivational introductory messages, send pre-survey text messages, and make multiple phone calls; however, the usefulness of all of these methods should be tested (Dal Grande et al., 2016, Gibson et al., 2019, L’Engle et al., 2018).

Overall, the IVR had a lower response rate than the GATS. Without a working sampling frame, assigning appropriate disposition codes, particularly distinguishing phone numbers that are active versus inactive, and calculating a response or cooperation rate is challenging. Our response rate is conservative in that phone calls that did not pick up (75.4 %) were labeled with the unknown disposition code. A certain percentage of these phone numbers are likely inactive, thereby being classified as ineligible, which would reduce the denominator for the response rate and increase its size (Phadnis et al., 2021; Yang Song et al., 2020). However, similar MPS in HICs yielded a higher response rate (Gundersen et al., 2014a, Gundersen et al., 2014b, Margo, 2012). For example, the 2012 Australian New South Wales Population Health Survey, an MPS, obtained about a 32 % response rate (Margo, 2012). Population-based surveys in LMICs (e.g., DHS) usually have a high response rate (United States Agency for International Development, 2021). Furthermore, BRFSS has a sampling frame of local working phone numbers (Centers for Disease Control and Prevention, 2017). Obtaining such a sampling frame of working mobile phone numbers is expensive and labor-intensive. Before collecting DHS data, a complete list of households is made in an enumeration area (United States Agency for International Development, 2021). A similar listing of working phone numbers may be made by randomly selecting enumeration areas; the same sampling frame could be used repeatedly for other surveys and may be updated regularly. In addition, differences in questionnaire design, survey length, and the calculation methods of disposition codes (i.e., break-offs, refusals, and partial interviews) may account for the differences in participation rates.

Our study has several notable strengths. We tested the reliability of our results by comparing them with a nationally representative survey, increasing the authenticity of our results. Our sample also included all mobile phone operators in Tanzania, removing any potential selection bias due to differences in subscribers’ characteristics between survey operators. As the data were collected anonymously, the risk of social desirability bias was additionally minimized.

However, limitations of the present study also warrant discussion. Though IVR had a large overall sample, the sample size in some age-sex strata was low, and we had the underrepresentation of some population groups. The information was collected based on self-reports and may be subject to recall bias. As the IVR sample only included participants with mobile phones, prevalence estimates for tobacco use among those without mobile phones are not known.

## 5 Conclusion

This study suggests that although there may be some differences in prevalence estimates obtained by IVR and household surveys, the point estimates could be close. There may be an underrepresentation of some population groups. Future studies should aim to increase the participation of people belonging to these groups. Additionally, the reliability of IVR findings should be tested in other LMICs.

## Funding

The study received finding from Bloomberg Phillantrophies.

## CRediT authorship contribution statement

**Gulam Muhammed Al Kibria:** Data curation, Writing – original draft, Writing – review & editing. **Frank Kagoro:** Writing – review & editing, Methodology, Investigation. **George Pariyo:** Writing – review & editing, Supervision, Project administration, Methodology, Investigation, Funding acquisition, Conceptualization. **Joseph Ali:** Writing – review & editing, Supervision, Methodology, Investigation. **Farida Hassan:** Writing – review & editing, Project administration, Data curation. **John W. Kilambo:** Writing – review & editing, Project administration, Investigation. **Irene Petro:** Writing – original draft, Project administration, Investigation. **Vidhi Maniar:** Writing – review & editing, Project administration, Methodology, Investigation. **Michelle R. Kaufman:** Writing – review & editing, Investigation. **Andres Vecino-Ortiz:** Writing – review & editing, Project administration, Methodology, Investigation. **Saifuddin Ahmed:** Writing – review & editing, Software, Resources, Project administration, Methodology, Investigation. **Honorati Masanja:** Writing – review & editing, Validation, Supervision, Resources, Project administration, Methodology, Investigation, Conceptualization. **Dustin G. Gibson:** Writing – review & editing, Writing – original draft, Validation, Supervision, Software, Resources, Project administration, Methodology, Investigation, Funding acquisition, Formal analysis, Conceptualization.

## Declaration of competing interest

The authors declare that they have no known competing financial interests or personal relationships that could have appeared to influence the work reported in this paper.

## Data Availability

Data will be made available on request.
