# Supplementary material for: A nationwide mobile phone survey for tobacco use in Tanzania: Sample quality and representativeness compared to a household survey
Source: Prev Med Rep. 2024 Jan 12;38:102609. doi: 10.1016/j.pmedr.2024.102609 (PMC10874872; doi:10.1016/j.pmedr.2024.102609)
Supplement: Supplementary data 1 [file mmc1.docx]

**S1 Table:**  Equations used to calculate survey rates Equation

| Analysis | Rate | Equation |
| --- | --- | --- |
| Primary | Contact Rate #1 | (I + P + R + O) / (I + P + R + O + NC + UH + UO) |
|  | Response Rate #2 | (I + P) / (I + P + R + O + NC + UH + UO) |
|  | Refusal Rate #1 | R / (I + P + R + O + NC + UH + UO) |
|  | Cooperation Rate #2 | (I + P) / (I + P +R + O) |

Abbreviations: I, interview; P, partial interview; R, refusal/break-off; O, other; NC, non-contact; UH, unknown household; UO, unknown other

**S2 Table: Questions from IVR and GATS**

| **Indicators** | **IVR** | **GATS** |
| --- | --- | --- |
| Current Smoker | I would now like to ask you about smoking tobacco, including cigarettes, cigars, shisha and pipes. Please do not include smokeless tobacco such as ugoro.  Do you currently smoke any tobacco products, such as cigarettes, cigars or pipes? If YES, press 1. If NO, press 3. | I would now like to ask you some questions about *smoking* tobacco, including cigarettes, cigars, pipes, shisha/hookar. Please do not answer about smokeless tobacco at this time.  Do you *currently* smoke tobacco on a daily basis, less than daily, or not at all?  Daily -- 1; Less than daily -- 2; Not at all -- 3; Don’t know -- 7; Refused -- 9 |
| Current Daily smoker | Do you currently smoke tobacco products daily? If YES, press 1. If NO, press 3. | **See above** |
| Former Smoker | In the past, did you ever smoke any tobacco products? If YES, press 1. If NO, press 3. | In the *past*, have you smoked tobacco on a daily basis, less than daily, or not at all?  Daily -- 1; Less than daily -- 2; Not at all -- 3; Don’t know -- 7; Refused -- 9 |
| Former Daily Smoker | In the past, did you ever smoke tobacco daily? If YES, press 1. If NO, press 3. | **See above** |
| Current smokeless tobacco user | The next question is about using smokeless tobacco including ugoro. Smokeless tobacco is tobacco that is not smoked but it is either chewed/sniffed.  Do you currently use any smokeless tobacco products such as ugoro? If YES, press 1. If NO, press 3. | The next questions are about using smokeless tobacco, such as snuff, chewing tobacco, pan, kuber. Smokeless tobacco is tobacco that is not smoked, but is sniffed through the nose, held in the mouth, or chewed.  Do you *currently* use smokeless tobacco on a daily basis, less than daily, or not at all?  Daily -- 1; Less than daily -- 2; Not at all -- 3; Don’t know -- 7; Refused -- 9 |
| Current daily smokeless tobacco user | Do you currently use smokeless tobacco products daily? If YES, press 1. If NO, press 3. | **See above** |
| Former smokeless tobacco user | In the past, did you ever use smokeless tobacco products? If YES, press 1. If NO, press 3. | Have you used smokeless tobacco daily in the past?  Yes – 1;No – 2; Don’t know –7; Refused -- 9 |
| Former daily smokeless tobacco user | In the past, did you ever use smokeless tobacco daily? If YES, press 1. If NO, press 3. | In the *past*, have you used smokeless tobacco on a daily basis, less than daily, or not at all?  Daily -- 1; Less than daily -- 2; Not at all -- 3; Don’t know -- 7; Refused -- 9 |

**S3 Table: Cost of conducting survey by age-sex strata**

| **To fill quota (n=385)** | **Phone calls** | **Pick-ups** | **Minutes of airtime** | **Quota filled** | **Complete Surveys** | **Calls/complete survey** | **Cost/complete survey** |
| --- | --- | --- | --- | --- | --- | --- | --- |
| Male 18-29 | 60,980 | 14,161 | 34,442 | 0 | 883 | 69 | $3.90 |
| Male 30-44 | 75,081 | 17,933 | 35,167 | 1154 | 634 | 118 | $5.55 |
| Female 18-29 | 6631 | 1838 | 3468 | 131 | 51 | 130 | $6.80 |
| Female 30-44 | 211,169 | 58,470 | 88,881 | 6385 | 573 | 368 | $15.51 |
| Males 45-59 | 92,965 | 21,320 | 29,392 | 3038 | 154 | 602 | $19.09 |
| No more quotas filled | 87,942 | 17,850 | 23,229 | 2514 | 67 | 1313 | $34.67 |
| **Total** | **534,768** | **131,572** | **214,579** | **13,222** | **2362** | **226** | **$9.08** |

Age-sex groups are presented in the order with which their quota (n=385) was filled.

**S4 Table: Comparison of tobacco use indicators by survey mode and age groups among males**

| **Variables** | **18-29** | | **30-44** | | **45-59** | | **≥60** | |
| --- | --- | --- | --- | --- | --- | --- | --- | --- |
|  | **IVR** | **GATS** | **IVR** | **GATS** | **IVR** | **GATS** | **IVR** | **GATS** |
| **Education level, % (n)** |  |  |  |  |  |  |  |  |
| No education | 5.8 (23) | 6.6 (41) | 8.7 (34) | 11.7 (85) | 6.6 (26) | 8.5 (29) | 13.7 (29) | 26.9 (76) |
| Primary | 44.4 (175) | 52.1 (322) | 51.5 (201) | 66.5 (482) | 57.5 (225) | 77.8 (267) | 43.6 (92) | 59.0 (167) |
| Secondary | 34.5 (136) | 36.1 (223) | 26.2 (102) | 14.9 (108) | 26.6 (104) | 9.9 (34) | 26.5 (56) | 11.0 (31) |
| Tertiary | 15.2 (60) | 5.2 (32) | 13.6 (53) | 6.9 (50) | 9.2 (36) | 3.8 (13) | 16.1 (34) | 3.2 (9) |
| **Place of residence, % (n)** |  |  |  |  |  |  |  |  |
| Urban | 60.9 (240) | 48.5 (300) | 56.7 (219) | 43.6 (316) | 48.6 (189) | 36.2 (124) | 45.8 (97) | 29.0 (82) |
| Rural | 39.1 (154) | 51.5 (318) | 43.3 (167) | 56.4 (409) | 51.4 (200) | 63.8 (219) | 54.2 (115) | 71.0 (201) |
| **Indicators, % (95% CI)** |  |  |  |  |  |  |  |  |
| Current smoker | 6.4 (4.4,9.3) | 9.4 (7.3,12.0) | 7.3 (5.1,10.3) | 19.3 (16.6,22.4)* | 13.7 (10.6,17.5) | 20.5 (16.5,25.1)* | 12.4 (8.6,17.6) | 25.9 (21.1,31.3)* |
| Current daily smoker | 2.6 (1.4,4.7) | 6.3 (4.7,8.6)* | 5.4 (3.6,8.2) | 15.1 (12.6,17.9)* | 12.4 (9.4,16.0) | 17.3 (13.6,21.6) | 9.0 (5.8,13.8) | 22.3 (17.8,27.6)* |
| Former smoker | 5.9 (3.9,8.7) | 5.7 (4.1,7.8) | 11.1 (8.4,14.7) | 10.1 (8.1,12.5) | 14.7 (11.5,18.6) | 14.3 (11.0,18.5) | 9.5 (6.2,14.3) | 19.1 (15.0,24.2)* |
| Former daily smoker | 1.5 (0.7,3.4) | 1.8 (1.0,3.2) | 5.7 (3.8,8.5) | 6.8 (5.1,8.8) | 8.2 (5.9,11.4) | 7.3 (5.0,10.6) | 6.7 (4.0,11.0) | 13.1 (9.6,17.6)* |
| Current smokeless tobacco user | 4.1 (2.5,6.6) | 1.6 (0.9,3.0)* | 2.1 (1.0,4.1) | 2.5 (1.6,3.9) | 4.4 (2.7,6.9) | 3.5 (2.0,6.1) | 5.7 (3.3,9.8) | 6.7 (4.3,10.3) |
| Current daily smokeless tobacco user | 1.5 (0.7,3.4) | 0.5 (0.2,1.5) | 1.6 (0.7,3.4) | 1.5 (0.8,2.7) | 2.8 (1.6,5.1) | 3.2 (1.8,5.7) | 3.3 (1.6,6.8) | 5.0 (3.0,8.2) |
| Former smokeless tobacco user | 3.1 (1.8,5.3) | 1.6 (0.9,3.0) | 6.0 (4.0,8.8) | 2.5 (1.6,3.9)* | 4.1 (2.5,6.6) | 2.9 (1.6,5.4) | 4.3 (2.2,8.1) | 3.2 (1.7,6.0) |
| Former daily smokeless tobacco user | 1.5 (0.7,3.4) | 0.5 (0.2,1.5) | 1.8 (0.9,3.8) | 0.8 (0.4,1.8) | 1.5 (0.7,3.4) | 0.3 (0.0,2.1) | 0.5 (0.1,3.3) | 2.1 (1.0,4.7) |

*: p <0.05, Abbreviations: CI: Confidence Interval, IVR: Interactive Voice Response, GATS: Global Adult Tobacco Survey

**S5 Table: Comparison of tobacco use indicators by survey mode and age groups among females**

| **Variables** | **18-29** | | **30-44** | | **45-59** | | **≥60** | |
| --- | --- | --- | --- | --- | --- | --- | --- | --- |
|  | **IVR** | **GATS** | **IVR** | **GATS** | **IVR** | **GATS** | **IVR** | **GATS** |
| **Education level, % (n)** |  |  |  |  |  |  |  |  |
| No education | 4.1 (16) | 13.0 (115) | 6.7 (26) | 17.5 (156) | 4.7 (6) | 20.3 (85) | 7.0 (5) | 62.9 (242) |
| Primary | 41.1 (160) | 54.4 (481) | 54.4 (210) | 68.6 (611) | 56.3 (72) | 72.2 (302) | 54.9 (39) | 33.8 (130) |
| Secondary | 36.2 (141) | 29.4 (260) | 27.2 (105) | 10.4 (93) | 26.6 (34) | 6.2 (26) | 31.0 (22) | 2.9 (11) |
| Tertiary | 18.5 (72) | 3.3 (29) | 11.7 (45) | 3.5 (31) | 12.5 (16) | 1.2 (5) | 7.0 (5) | 0.5 (2) |
| **Place of residence, % (n)** |  |  |  |  |  |  |  |  |
| Urban | 62.1 (242) | 44.5 (394) | 60.3 (232) | 40.8 (364) | 58.3 (74) | 35.6 (149) | 52.1 (37) | 23.8 (93) |
| Rural | 37.9 (148) | 55.5 (491) | 39.7 (153) | 59.2 (528) | 41.7 (53) | 64.4 (270) | 47.9 (34) | 76.2 (297) |
| **Indicators, % (95% CI)** |  |  |  |  |  |  |  |  |
| Current Smoker | 1.3 (0.5,3.1) | 0.2 (0.1,0.9)* | 2.3 (1.2,4.4) | 0.9 (0.4,1.8)* | 1.6 (0.4,6.1) | 1.7 (0.8,3.5) | 4.2 (1.4,12.4) | 4.6 (2.9,7.2) |
| Current Daily smoker | 0.3 (0.0,1.8) | 0.1 (0.0,0.8) | 0.8 (0.3,2.4) | 0.8 (0.4,1.6) | 0.8 (0.1,5.4) | 1.4 (0.6,3.2) | 0.0 (.,.) | 3.3 (1.9,5.7) |
| Former Smoker | 1.8 (0.9,3.8) | 0.8 (0.4,1.7) | 2.3 (1.2,4.4) | 1.0 (0.5,1.9) | 1.6 (0.4,6.1) | 1.7 (0.8,3.5) | 1.4 (0.2,9.5) | 5.6 (3.7,8.4) |
| Former Daily Smoker | -- | -- | 0.5 (0.1,2.1) | 0.7 (0.3,1.5) | 0.0 (.,.) | 1.0 (0.4,2.5) | 0.0 (.,.) | 3.8 (2.3,6.3) |
| Current smokeless tobacco user | 1.6 (0.7,3.4) | 0.3 (0.1,1.1)* | 2.9 (1.6,5.1) | 1.1 (0.6,2.1)* | 5.5 (2.6,11.2) | 3.3 (2.0,5.6) | 7.0 (2.9,15.9) | 13.6 (10.5,17.4) |
| Current daily smokeless tobacco user | 0.3 (0.0,1.8) | 0.1 (0.0,0.8) | 1.3 (0.5,3.1) | 0.8 (0.4,1.6) | 2.4 (0.8,7.1) | 2.4 (1.3,4.4) | 2.8 (0.7,10.7) | 11.8 (8.9,15.4)* |
| Former smokeless tobacco user | 2.3 (1.2,4.4) | 0.0 (.,.) | 0.8 (0.3,2.4) | 0.8 (0.4,1.6) | 0.0 (.,.) | 1.7 (0.8,3.5) | 0.0 (.,.) | 7.9 (5.6,11.1) |
| Former daily smokeless tobacco user | 0.3 (0.0,1.8) | 0.0 (.,.) | 0.3 (0.0,1.8) | 0.3 (0.1,1.0) | 0.0 (.,.) | 0.5 (0.1,1.9) | 0.0 (.,.) | 4.9 (3.1,7.5) |

*: p <0.05, Abbreviations: CI: Confidence Interval, IVR: Interactive Voice Response, GATS: Global Adult Tobacco Survey
